# Atomic Manipulation of 2D Materials by Scanning Tunneling Microscopy: Advances in Graphene and Transition Metal Dichalcogenides

**DOI:** 10.3390/nano15120888

**Published:** 2025-06-08

**Authors:** Tingting Wang, Lingtao Zhan, Teng Zhang, Yan Li, Haolong Fan, Xiongbai Cao, Zhenru Zhou, Qinze Yu, Cesare Grazioli, Huixia Yang, Quanzhen Zhang, Yeliang Wang

**Affiliations:** 1 School of Integrated Circuits and Electronics & Yangtze Delta Region Academy, Beijing Institute of Technology (BIT), Beijing 100081, China; tingting.wang@bit.edu.cn (T.W.); zhanlingtao02@163.com (L.Z.); li@bit.edu.cn (Y.L.); 3120231569@bit.edu.cn (H.F.); 1120210142@bit.edu.cn (X.C.); 3120241396@bit.edu.cn (Z.Z.); 1120210309@bit.edu.cn (Q.Y.); yanghuixia@bit.edu.cn (H.Y.); 2 CNR—Istituto Officina dei Materiali (IOM), S.S. 14 km 163.5, 34149 Trieste, Italy; grazioli@iom.cnr.it

**Keywords:** scanning tunneling microscopy (STM), atomic manipulation, graphene, transition metal dichalcogenides (TMDs)

## Abstract

This review provides a comprehensive overview of recent advances in atomic-scale manipulation of two-dimensional (2D) materials, particularly graphene and transition metal dichalcogenides (TMDs), using scanning tunneling microscopy (STM). STM, originally developed for high-resolution imaging, has evolved into a powerful tool for precise manipulation of 2D materials, enabling translational, rotational, folding, picking, and etching operations at the nanoscale. These manipulation techniques are critical for constructing custom heterostructures, tuning electronic properties, and exploring dynamic behaviors such as superlubricity, strain engineering, phase transitions, and quantum confinement effects. We detail the fundamental mechanisms behind STM-based manipulations and present representative experimental results, including stress-induced bandgap modulation, tip-induced phase transformations, and atomic-precision nanostructuring. The versatility and cleanliness of STM offer unique advantages over conventional transfer methods, paving the way for innovative applications in nanoelectronics, quantum devices, and 2D material-based systems. Finally, we discuss current challenges and future prospects of integrating STM manipulation with advanced computational techniques for automated nanofabrication.

## 1. Introduction

The discovery of graphene in 2004 ignited widespread interest in two-dimensional (2D) materials and their promising applications in next-generation technologies [1]. These materials, composed of monolayers or a few of layers of atomic structures, possess a thickness much smaller than their lateral dimensions [2,3]. Within individual layers, atoms are strongly bonded via covalent interactions, while adjacent layers are held together by weak van der Waals (vdW) forces [4,5]. Due to their unique structural and electronic properties, 2D materials have demonstrated significant potential in electronics [6,7,8,9], optoelectronics [10,11,12,13], and various emerging fields [14,15,16,17,18,19,20,21], positioning them as strong candidates for extending Moore’s Law.

**Figure 1:**
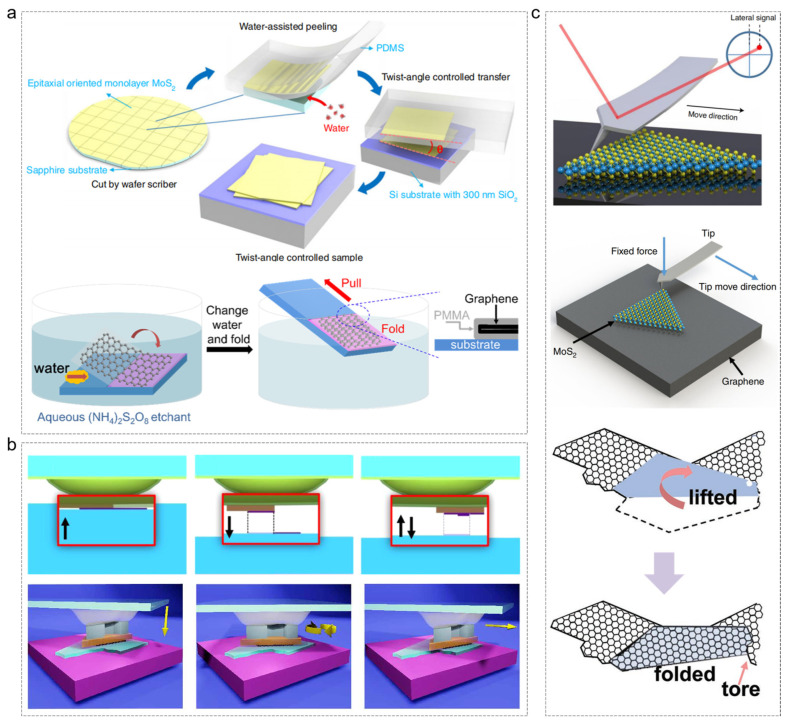
Schematic diagrams of three clean construction methods of 2D materials. (**a**) Liquid-phase assisted exfoliation [22,23]. (**b**) Mechanical exfoliation [24,25]. (**c**) Scanning probe microscopy (SPM) manipulation [14,27,28]. (**a**) Reprinted with permission from Ref. [22] from Liao, M. et al., copyright 2020, and Ref. [23] from the American Chemical Society, copyright 2017. (**b**) Reprinted with permission from Ref. [24] from the American Chemical Society, copyright 2016, and Ref. [25] from The American Association for the Advancement of Science, copyright 2020. (**c**) Reprinted with permission from Ref. [14] from Liao, M. et al., copyright 2021, Ref. [27] from Liao, M. et al., copyright 2017, and Ref. [28] from WILEY-VCH Verlag GmbH & Co. KGaA, Weinheim, copyright 2018.

Efficient construction techniques are essential for the reliable characterization and integration of 2D materials into functional devices. The degree of cleanliness achieved during construction directly impacts the stability and reproducibility of their intrinsic properties. Currently, three primary clean construction methodologies are employed—liquid-phase assisted exfoliation [22,23], mechanical transfer [24,25], and scanning probe microscopy (SPM)-based manipulation [14,26,27,28]—along with several other emerging techniques [29]. Liquid-phase assisted exfoliation (Figure 1a) facilitates large-scale material preparation via solution-based processes, but often results in solvent or polymer residues, compromising interfacial purity. Mechanical exfoliation (Figure 1b) enables angular control through prefabricated patches, yet it is constrained by limited material size, precision, and dependency on intermediary layers. In contrast, SPM (Figure 1c) distinguishes itself by operating in dry, ultrahigh-vacuum environments, allowing for direct, contamination-free manipulation of materials at the atomic scale.

SPM offers dual capabilities: high-resolution imaging and precise manipulation of nanoscale materials. Unlike traditional transfer methods, SPM enables the dynamic, controlled positioning of 2D materials, critically influencing properties such as superlubricity [5], superlattice formation [30], and even superconductivity [31]. Its ability to ensure atomic-level cleanliness, combined with exceptional spatial control, makes SPM an ideal platform for the high-precision transfer and performance tuning of 2D materials. In SPM operations, AFM and STM techniques are commonly employed. These two methods are applied in different scenarios. STM utilizes current feedback to acquire sample topography and electronic properties, while AFM primarily relies on force feedback to obtain sample morphology and chemical bonding information. Both techniques can accomplish the structuring of target materials. However, when conducting research on electronic states and conductive properties of two-dimensional materials, the STM manipulation technique demonstrates greater advantages.

This review focuses on five prevalent scanning tunneling microscopy (STM)-based techniques for manipulating 2D materials—primarily graphene and transition metal dichalcogenides (TMDs)—including translation, rotation, folding, picking, and etching. We provide detailed examples of structural modifications achieved through these methods, addressing critical aspects such as interlayer spacing, strain engineering, phase transitions, and the fabrication of custom nanostructures. Finally, we discuss current limitations and outline future directions for the development and application of STM manipulation in advanced material research.

## 2. STM Manipulation

Scanning probe microscopy (SPM) has revolutionized nanoscience by enabling not only high-resolution imaging but also the precise manipulation of matter at the nanoscale, even down to the individual atoms. SPM uses a sharp tip to probe local surface properties with nanometer or atomic resolution, allowing for the generation of real-space, high-resolution images [32].

Among SPM techniques, STM, developed by Binnig and Rohrer in 1982 [33], has been particularly influential, earning them the Nobel Prize in Physics in 1986. In an STM experiment, a bias voltage is first applied between the sample and the probe. When the distance between the probe and the sample decreases to approximately a few nanometers, a tunneling current caused by the quantum tunneling effect can be detected. Since the tunneling current exhibits an exponentially decaying relationship with the separation between the sample and the tip, the scanning tunneling microscope is highly sensitive to minute topographic variations on the surface. By scanning the tip across the entire sample, the topographic information of the sample surface can be obtained.

### 2.1. STM Manipulation in Zero-Dimensional and One-Dimensional Materials

**Figure 2:**
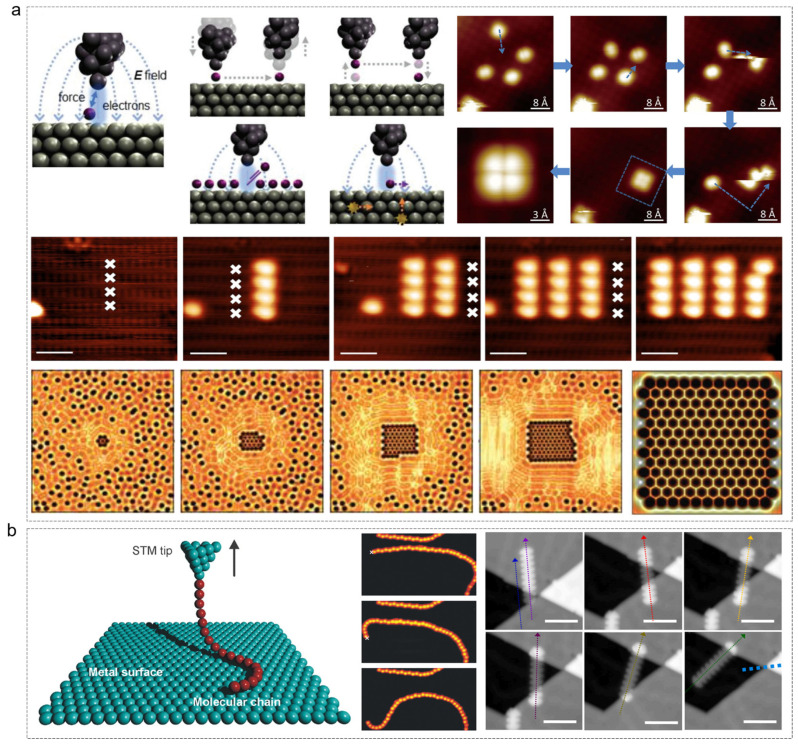
STM manipulation application in single-molecule, multi-molecule, and 1D molecular chains. (**a**) Tip–atom interaction and experimental schemes [38] and application for atom manipulation [39,40,41]. The dotted lines with arrows indicate that the blue dotted lines marked in the figure represent the movement trajectory of the molecules, and the white “×” represent the coordinates of the hydro-gen atoms. (**b**) Tip–atom experimental schemes [42] and application for 1D molecular chains [42,43]. The colored dotted lines with arrows indicate the linear outline of the molecule, and the cyan dotted line indicates the relative position of gold to the molecules. (**a**) Reprinted with permission from Ref. [38] from WILEY-VCH Verlag GmbH & Co. KGaA, Weinheim, copyright 2019, Ref. [39] from Moller, M. et al., copyright 2017, Ref. [40] from Springer Nature Limited, copyright 2012 and Ref. [41] from Springer Nature Limited, copyright 2014. (**b**) Reprinted with permission from Ref. [42] from The American Association for the Advancement of Science, copyright 2009 and Ref. [43] from Elsevier B.V. All rights reserved, copyright 2022.

The exploration of novel physical properties through dimensional reduction—from three-dimensional (3D) bulk materials to two-dimensional (2D) graphene [34], one-dimensional (1D) carbon nanotubes [35], and zero-dimensional (0D) fullerenes [36] or quantum dots [37] has greatly expanded the frontiers of nanoscience. STM has played a pivotal role in enabling the precise manipulation of 0D molecules (as shown in Figure 2a) [38,39,40,41] and 1D molecular chains (as shown in Figure 2b) [42,43], facilitating atomic-level control.

A landmark achievement was demonstrated by Eigler et al., who arranged Xe atoms on a Ni (110) surface to spell “IBM” with atomic precision [44]. STM has also been used for vertical manipulation, such as transferring CO molecules [45] and Ca dimer clusters [46] between the tip and substrate, and has been employed for on-surface chemical reactions, such as the Ullmann coupling [47]. For 1D systems, STM has been used to construct metal atomic chains [48], spin chains [49], and quantum confinement structures [50]. Moreover, STM allows for the guided alignment of molecular chains, such as CO on Cu (111) to form “molecular graphene” [40], and iodobenzene chains [51]. It further facilitates the fabrication of 1D heterojunctions, such as graphene nanoribbons [52] and semiconductor atomic lines [53].

### 2.2. STM Manipulation Methods in Two-Dimensional Materials

The STM-based manipulation of 2D materials predominantly involves five distinct techniques: translation [26,54,55,56,57], rotation [26,54,55,56,57], folding [57,58,59,60], picking [61,62,63], and cutting [64,65]. Successful execution of these operations depends critically on achieving ultralow friction between the 2D material and the substrate, a condition associated with superlubricity [16,19,56]. These manipulation strategies enable precise control over both the spatial positioning and geometric configuration of 2D materials.

#### 2.2.1. Translation and Rotation

**Figure 3:**
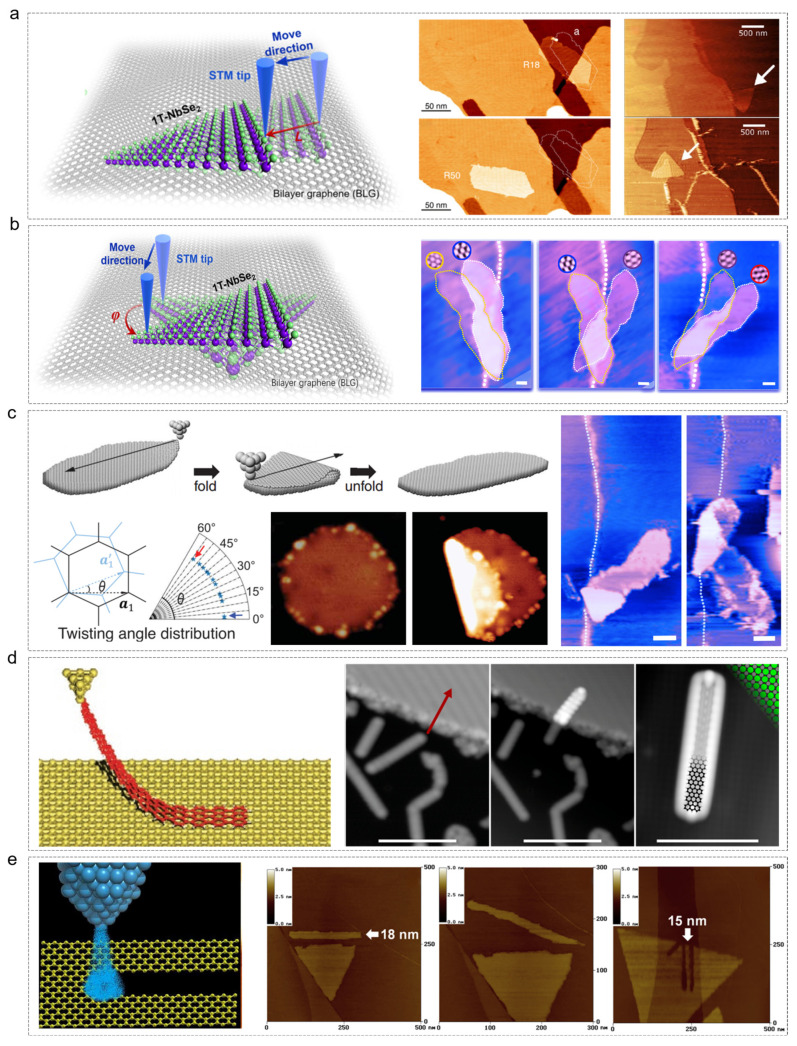
Five kinds of STM manipulation method. Experimental schemes and applications for (**a**) transition [54,55,56], (**b**) rotation [54,57] (**c**) folding [57,58] (**d**) picking [61,62], and (**e**) cutting [64,65]. The red arrow in (d) indicates the movement trajectory of the molecule. (**a**) Reprinted with permission from Ref. [54] from the American Chemical Society, copyright 2022, Ref. [55] from the American Chemical Society, copyright 2014, and Ref. [56] from Büch, H. et al., copyright 2018. (**b**) Reprinted with permission from Ref. [54] from the American Chemical Society, copyright 2022, and Ref. [57] from the American Physical Society, copyright 2022. (**c**) Reprinted with permission from Ref. [57] from the American Physical Society, copyright 2022, and Ref. [58] from The American Association for the Advancement of Science, copyright 2019. (**d**) Reprinted with permission from Ref. [61] from Springer Nature, copyright 2012, and Ref. [62] from the American Chemical Society, copyright 2018. (**e**) Reprinted with permission from Ref. [64] from Springer Nature, copyright 2014, and Ref. [65] from Elsevier, copyright 2016.

The ability to translate and rotate 2D materials hinges on minimizing interfacial friction [14,66,67]. When there is significant lattice mismatch between the material and substrate, friction is reduced, facilitating easier manipulation. However, when the lattices are commensurate, as in the case of graphene flakes on graphite [16,68], higher forces are required to overcome the increased friction. The practical examples of translation [54,55,56] and rotation [54,57] are shown in Figure 3a and Figure 3b, respectively. For example, 1T-NbSe_2_ islands grown on bilayer graphene (BLG) can be manipulated via STM tip [54]. The tip is guided along a specific trajectory to the island’s edge, where the repulsive force between the tip and 1T-NbSe_2_ island enables controlled sliding. The STM manipulation protocol requires precise positioning of the scanning probe tip within the quantum tunneling regime (typically maintained at 1 nA tunneling current under −0.1 V bias voltage). The tip is then translated along predefined trajectories with atomic-scale precision, employing a controlled scanning rate below 1 nm/s. During this process, the tip-induced repulsive forces generated in the noncontact tunneling configuration enable controlled displacement of islands by overcoming their interfacial friction with the substrate. Translation distance (L) and rotation angle (φ) can be monitored in real time using atomic-level STM images.

#### 2.2.2. Folding (Origami)

Folding, or nanoscale origami, increases the effective thickness of 2D materials and enables the creation of complex three-dimensional nanostructures as shown in Figure 3c [57,58]. This technique requires the material to be highly flexible—an inherent property of many 2D systems. The process involves initially placing the STM tip on the edge of the 2D materials and gradually reducing the vertical distance between them. This causes an increase in the electrostatic force between the STM tip and the edge. By moving the tip in a specific direction, the edge of the 2D island is lifted and shifted accordingly. The manipulation process involves precisely positioning the STM tip in proximity to the edge of a graphene nanostructure through controlled tip–substrate distance reduction. When the induced van der Waals forces between the tip and nanostructure surpass the interfacial adhesion force binding the nanostructure to the substrate, the tip initiates edge-selective detachment. This enables controlled directional transport of the nanostructure along a predetermined tip trajectory through continuous van der Waals interaction modulation. The process concludes with tip retraction and precise deposition of the relocated nanostructure segment at targeted coordinates, completing the nondestructive nanoscale manipulation cycle. Eventually, the lifted portion is folded onto another part of the graphene, giving rise to graphene-folded nanostructures. Within these intricate nanostructures, the lifted graphene sheet forms a flat stacking structure with the remaining sheets, while a partially closed tubular structure emerges at the connection point. Likewise, if the tip is positioned on the edge of the folded section within the folded graphene nanostructure and the vertical distance between the tip and the edge is gradually reduced, the folded part can be lifted once again by the STM tip and placed back onto the graphite surface. This restores the state of the graphene island prior to folding. This folding and unfolding process of graphene nanoislands can be repeatedly reversed in any direction.

#### 2.2.3. Picking and Transfer

STM can also facilitate the pickup and transfer of nanostructures across steps or onto different substrates, like Figure 3d [61,62]. This method relies on van der Waals or chemical bonding between the tip and the material, forming a suspended junction. The target material to be picked up must be a single crystal or of high crystallinity (e.g., graphene grown by chemical vapor deposition) to avoid defects that could lead to failure or distort transport properties. Typically, the material’s length is less than 50 nm to prevent breakage due to mechanical stress. Narrow-band materials are more likely to form stable suspended junctions. The process begins by confirming the target material’s position and edge structure (e.g., zigzag or armchair type) using atomic resolution STM images. The specific endpoints of the material (such as the zigzag edge) are selected for contact to enhance binding via edge-localized states. The STM tip is then slowly lowered to the material surface, and contact is judged by a current jump (e.g., a sudden decrease from 1 nA to 0.1 nA). Subsequently, the tip is retracted at a constant speed (0.1–1 nm/s), and the current–distance (I–z) curve is monitored. If the current decays exponentially with distance (β ≈ 0.1–0.8 Å^−1^), it indicates that the material has been successfully picked up and a suspended junction has been formed.

#### 2.2.4. Cutting and Etching

STM-based nanofabrication employs tunneling electrons to induce localized etching, enabling precise cutting of graphene and other 2D lattices [65]. The core mechanism involves high-energy tunneling electrons breaking the carbon–carbon bonds (Figure 3e), with the assistance of oxygen or water molecules in the environment, enabling atomic-level edge control. Atomic-resolution images at low bias (5–50 mV) and tunneling currents (1–2 nA) are used to determine the zigzag and armchair crystallographic directions. To induce etching, a negative bias of 2.0–2.3 V is applied to activate the tunneling electron beam. The tip is moved at a speed of 1–5 nm/s along the predefined direction, ensuring continuous cutting. This process is carried out in an atmosphere with humidity levels of 70–75%, where the oxidizing effect of water molecules facilitates the etching reaction.

## 3. STM Manipulation Application

Atomic-scale manipulation using STM has become indispensable in the study of 2D materials. This technique offers a flexible, contamination-free, and highly precise method for controlling nanoscale structures. As the family of 2D materials continues to grow, research has moved beyond static structural analysis towards the exploration of dynamic, tunable, and multi-dimensional material properties. STM tip-based manipulation provides unique opportunities to investigate and engineer complex behaviors, including electronic coupling, strain effects, phase transitions, and custom nanostructure fabrication.

Apart from altering the electrical properties of materials by positioning the sample, STM techniques can also be utilized to “write” and “read” spin states. [69] Using two STM manipulation approaches—electron-induced dehydrogenation and tip–nanostructure bonding—controllable switching of local spin states in graphene nanostructures has been achieved [70].

### 3.1. Distance Regulation Between Islands

STM enables the systematic study of electronic phenomena that emerge from varying the spacing between nanoscale islands, such as 1D correlated electronic properties [71,72,73]. By combining atomic-scale imaging with spectroscopic capabilities, STM can precisely characterize both structural morphology and local electronic states as a function of inter-island distance.

**Figure 4:**
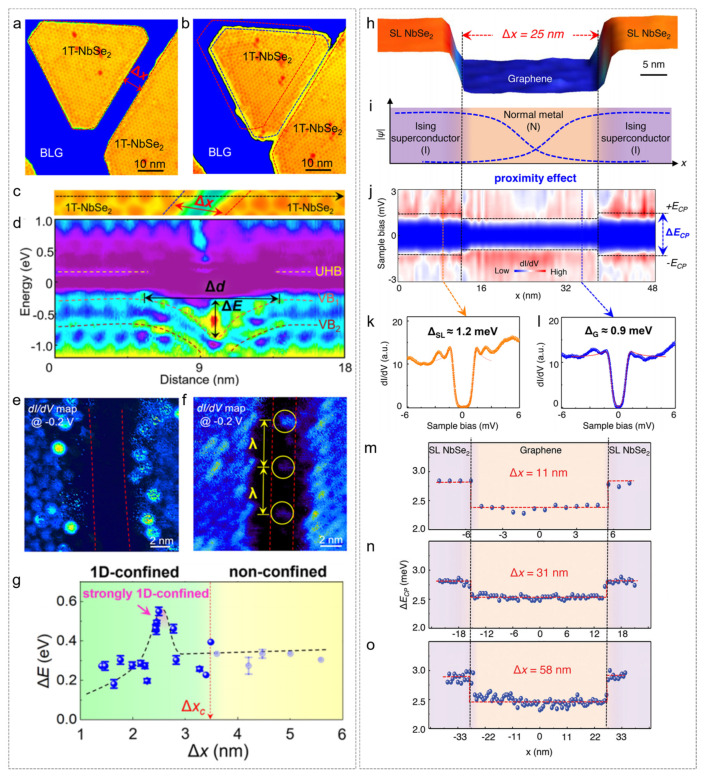
The specific application of STM tip control distance. (**a**–**g**) Two T-NbSe_2_ on BLG with different interspacing [54]. (**a**,**b**) STM images with large and small distances, respectively. (**c**) Zoomed-in STM images of the panels (**b**). (**d**) Spatially resolved d*I*/d*V* spectra marked by the black arrows in the panels (**c**). (**e**,**f**) d*I*/d*V* maps at the bias of −0.2 V recorded with Δx ≈ 3.4 nm and 2.5 nm, respectively. (**g**) ΔE as a function of Δx. Δx: the interspacing between the two islands. ΔE: the depth of the 1D-confined electron potential. (**h**–**o**) Two H-NbSe_2_ on BLG with different interspacing [74]. (**h**) Typical STM image of single-layer NbSe_2_ islands on graphene. (**i**) Schematic superconducting order of the INI junctions. (**j**) Spatially resolved STS spectra recorded across the junction. (**k**,**l**) Typical STS spectra recorded at the NbSe_2_ and graphene, respectively. (**m**–**o**) Spatially resolved energy separations of the two coherent peaks E_cp_ in the superconducting spectra of graphene. The distances (Δx) of the two adjacent island edges are 11, 31, and 58 nm, respectively. (**a**–**g**) Reprinted with permission from Ref. [54] from the American Chemical Society, copyright 2022. (**h**–**o**) Reprinted with permission from Ref. [72] from Advanced Quantum Technologies published by Wiley—VCH GmbH, copyright 2023.

Zhang et al. demonstrated that the electronic properties of two 1T-NbSe_2_ islands change dynamically as their separation decreases [54]. When the islands are far apart (Figure 4a), no electronic states are observed in the intervening region, as observed in the corresponding d*I*/d*V* map. However, as the islands approach each other, the apparent depth of the homojunction became shallower, and the band bending in the vicinity of the islands also decreased, with the speed of the gap reduction accelerating as the distance between the islands decreased (Figure 4b, with a gap of 2.4 nm). The atomic-resolution image and d*I*/d*V* line spectra (Figure 4c,d) showed that the energy bands between the islands bent downward as the spacing reduced.

Moreover, the superconducting properties of single-layer H-NbSe_2_ islands exhibit a significant size dependence, with a critical area of approximately 800 nm^2^ [74]. When the island area (S) exceeds this critical value, scanning tunneling spectroscopy (STS) reveals particle–hole symmetric coherence peaks, indicating the presence of a uniformly distributed superconducting gap (approximately 1.2 meV), with the gap extending to the island’s edge. However, when S < 800 nm^2^, the superconductivity is suppressed and transitions to insulating behavior, likely due to Coulomb gaps induced by quantum confinement effects.

When such superconducting islands form an Ising superconductor–normal metal-Ising superconductor (INI) Josephson junction with graphene, a spatially uniform superconducting gap (approximately 0.9 meV) can be induced in graphene. Experiments confirm that even when the distance (Δx) between NbSe_2_ islands is artificially expanded to 150 nm, graphene still maintains a superconducting gap, with only a slight decay at the center, which contrasts sharply with the much smaller decay length observed in conventional NbSe_2_–metal junctions. This is likely due to the enhanced proximity effect from multiple Andreev reflections at both interfaces. This macroscopic superconducting phenomenon induced by a high-density array of NbSe_2_ islands provides a new avenue for the design of graphene-based quantum devices.

### 3.2. Strain Regulation

Mechanical strain offers a powerful lever to modulate the physical, chemical, and electronic properties of 2D materials, enabling the design of tunable functional devices. STM manipulation provides nanoscale control over strain application.

**Figure 5:**
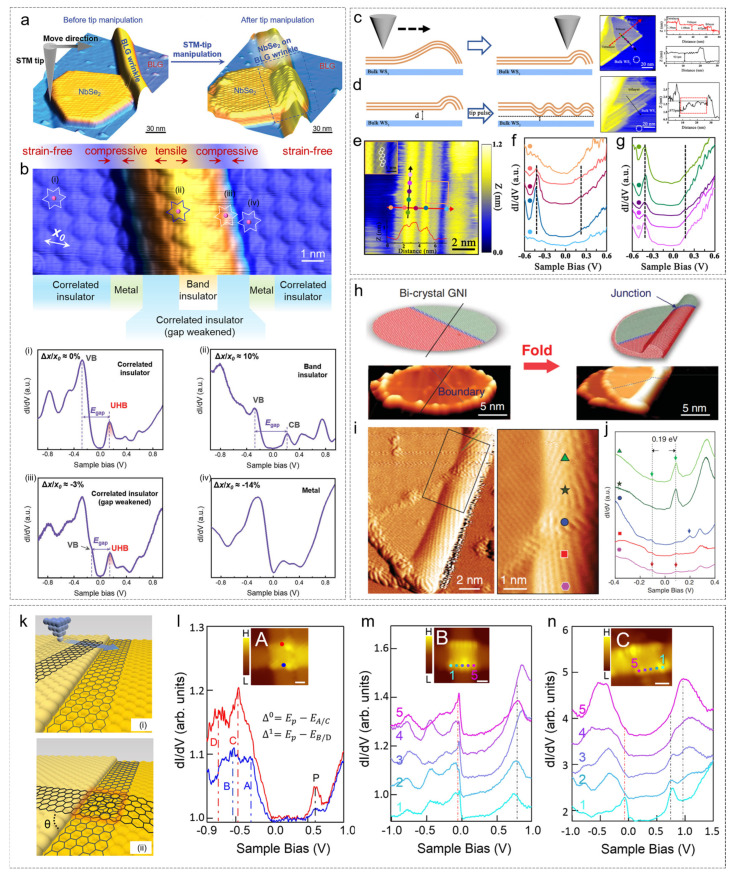
Strain regulation via STM manipulation technique. (**a**,**b**) Nanoscale control of a single-layer T-phase NbSe_2_ island on and off the graphene wrinkle via an in situ STM manipulation technique [75]. (**a**). STM images before and after the manipulation. The white frame represents star-of-David and the central niobium atom is depicted by a pink dot. (**b**) The topographic image and electronic structures of the island on the graphene wrinkle. (i–iv) STS spectra acquired in panel (**b**). (**c**–**g**) STM manipulation for flattening and restoring graphene nanoscale wrinkles (GNWs). [76] (**c**,**d**). A schematic diagram and STM images of flattening and restoring GNWs using the STM tip method. (**e**) An STM image of a reconstructed GNW; (**f**) d*I*/d*V* spectra taken at different positions across the GNW in (**e**) along the red arrow. (**g**) d*I*/d*V* spectra taken at different positions across the GNW in (**e**) along the black arrow. (**h**–**j**) Folding a graphene nanoisland (GNI) [58]. (**h**) Schematic diagram (top) and STM image (bottom) before and after folding a bicrystal GNI. (**i**) (Left) Large-scale view and (right) zoom-in image of the black rectangular area at left. (**j**) The d*I*/d*V* spectra recorded along the GNW labeled by the color and symbols in (**i**). (**k**) Schematic diagrams of zigzag GNRs before and after STM tip manipulation, respectively [76]. (**l**–**n**) STS taken at the zigzag edges of three twisted bilayer zigzag GNR junctions [76]. Insets in (**l**–**n**) are the STM images of the three junctions indicating the STS were taken. The positions on the spectrum are distinguished by different colors, and the corresponding spectral lines are displayed in the same color. (**a**,**b**) Reprinted with permission from Ref. [75] from Advanced Science published by Wiley—VCH GmbH, copyright 2023. (**c**–**g**) Reprinted with permission from Ref. [76] from the American Physical Society, copyright 2022. (**h**–**j**) Reprinted with permission from Ref. [58] from The American Association for the Advancement of Science, copyright 2019. (**k**–**n**) Reprinted with permission from Ref. [58] from Wang, D. et al., copyright 2023.

Hou et al. manipulated the T-phase NbSe_2_ islands grown on BLG using STM tip control, moving the islands from the wrinkle side to the wrinkle itself (Figure 5a) [75]. It was observed that the NbSe_2_ islands at different positions on the wrinkle experienced varying degrees of tensile and compressive stress. This stress caused changes in the bandgap of the insulating NbSe_2_ phase. In region (ii) of Figure 5b under tensile stress, the NbSe_2_ bandgap broadened, transforming from a Mott insulator to a band insulator. In region (iii) of Figure 5b, under compressive stress, the bandgap was compressed, and as the compression increased, the gap gradually decreased until it became metallic, as shown in region (iv) of Figure 5b.

Guan et al. transferred few-layer graphene onto bulk WS_2_ substrates and used STM tip manipulation to control the graphene wrinkles, investigating the effect of stress on the graphene bandgap [76]. The manipulation methods are shown in Figure 5c,d. In Figure 5c, the STM image shows the morphology after the wrinkles were smoothed by the STM tip. The height profile on the right indicates that the graphene was flattened, with only one remaining wrinkle. The tip pulse method was then used to reconstruct graphene nanowrinkles, reducing the distance between the wrinkle valley and the substrate, enhancing the coupling between graphene and WS_2_, and opening the graphene bandgap. A metal–semiconductor–metal structure was constructed on the graphene nanosheet.

From the above experiments, it is evident that stress engineering provides an efficient way to open the graphene bandgap. In 2019, Chen et al. used STM tip manipulation to fold graphene on a HOPG substrate [58]. This folding was repeatable and could be carried out at arbitrary angles. By selecting the folding direction, they could customize the semiconductor or metallic tube-like edges. In addition to folding graphene sheets, they also folded bicrystalline graphene nanodisks with 5–7 ring grain boundaries. After folding, the defects in the original plane evolved into molecular heterojunctions (IMJs) connecting two different chiral carbon nanotubes (CNTs). The van Hove singularity (VHS) bandgap of both segments of the tubes was 0.19 eV, indicating that their semiconducting properties were mainly determined by the tube diameter.

Further, in 2020, Chen et al. successfully controlled the twist angle of bilayer graphene using the STM tip manipulation method with a configuration of a periodic alternation of pentagons and heptagons grain boundary [77]. Later, in 2022, they used the STM tip to construct bilayer zigzag graphene nanoribbons (6-GNR) with a specific twist angle (θ) on a Au (111) surface [78]. Experimental and theoretical studies revealed that two stacked 6-GNRs with the same twist angle (θ = 90°) exhibited three different stacking offsets in the twisted bilayer zigzag GNR junction: Model A (high-symmetry stacking), Model B (low-symmetry stacking), and Model C (asymmetric stacking). The edge states of these three stacking configurations were significantly different. In Model A, the STS spectrum showed a clear bandgap at the edges, which was significantly smaller than the bandgap of a single-layer zigzag GNR (Δ^0^ = 1.5 eV). No near-zero energy peak was observed, indicating that flat bands did not form under high-symmetry stacking. In Model B, the near-zero energy peak expanded, corresponding to the formation of flat bands, and the bandgap further reduced, indicating stronger interlayer coupling. In Model C, a near-zero energy peak was observed only at the junction corner (point I), rapidly decaying towards the opposite corner (point S), as shown by the red dashed line.

### 3.3. Phase Transformation Induced by STM

STM tip-induced voltage pulses can drive local phase transitions in 2D materials, offering a direct method to engineer electronic phases with spatial precision.

In 1996, Zhang et al. first demonstrated STM-induced phase transition in TaSe_2_ [79]. In 2017, Bischof et al. later triggered a local phase transition of NbSe_2_ from the 2H phase to the 1T phase by applying voltage pulses greater than 4 V (lasting 100 ms) [80]. Additionally, using low-temperature STM, they performed reversible phase transitions on the surface of NbSe_2_ at high-bias voltages (5–6 V). When the STM tip repeatedly scanned with a 5–6 V high bias, part of the 3 × 3 charge density wave (CDW) region transformed into a 1D CDW. After lowering the bias and scanning again, the 1D CDW region reverted to the original 3 × 3 CDW structure, demonstrating the reversibility of the phase transition. The high-bias voltage from the STM tip may induce lattice strain via localized Joule heating or charge injection, breaking the symmetry of the 3 × 3 CDW and promoting its transformation into a strain-stabilized 1D-CDW. In addition to modulating the period of the CDW in the sample, Song et al. also successfully manipulated the hand phase of the CDW in T-phase NbSe_2_ via tip-induced pulses [81].

In a 2024 study, the authors explored the voltage pulse-induced 1T’→1T structural phase transition in monolayer FeSe_2_ at various temperatures [82]. They investigated the threshold voltage and reversibility of the phase transition at temperatures ranging from 5 K to 60 K. The study found that as the temperature increased, the threshold voltage for the phase transition increased. Above 50 K, the 1T phase spontaneously reverted to the 1T’ phase. At 60 K, 54% of the islands could not be converted by the pulse. Ren et al. utilized STM voltage pulses to induce local mechanical fractures and phase transitions at the interface of graphene–WSe_2_ heterostructures, achieving the transformation of WSe_2_ from the 1H phase to the 1T’ phase [83].

**Figure 6:**
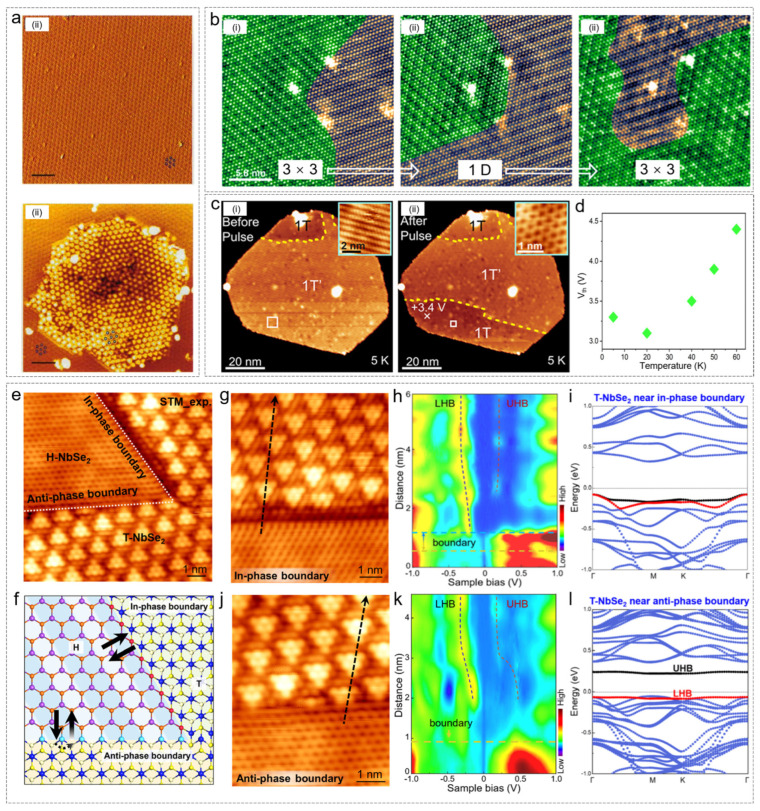
STM tip voltage pulse-induced phase transition. (**a**) STM images characterized the phase transformation of TaSe_2_ from 2H to the 1T [79]. (**b**) Tip-induced transitions of NbSe_2_ between the 1D-CDW and the 3 × 3 CDW [80]. (**c**) Electrically induced structural phase transition in FeSe_2_ monolayers [82]. (**d**) Green rhombuses stand for average threshold voltage Vth at different temperatures. (**e**–**l**) Tip electrical pulse-induced transitions of NbSe_2_ from the H phase to the T phase [84]. (**e**) STM image after applying a tip pulse. (**f**) The model diagram corresponding to (**e**). (**g**,**j**) Atomically resolved STM image of in-phase and anti-phase boundary, respectively. (**h**,**k**) Spatially resolved STS spectra measured across the in-phase and anti-phase boundary along the black dotted arrow in panel (**g**) and panel (**j**), respectively. (**i**,**l**) Calculated band structures near the in-phase and anti-phase boundaries, respectively. (**a**) Reprinted with permission from Ref. [79] from The American Association for the Advancement of Science, copyright 1996. (**b**) Reprinted with permission from Ref. [80] from the American Chemical Society, copyright. (**c**) Reprinted with permission from Ref. [82] from the American Chemical Society, copyright 2024. (**e**–**l**) Reprinted with permission from Ref. [84] from the American Chemical Society, copyright 2025.

In a more detailed study by Chen et al. in 2025, the researchers observed two types of boundaries after applying voltage pulses based on whether the selenium atoms were aligned in the same direction [84]. Boundaries with selenium atoms aligned along the same line were termed “same-direction”, while those with misaligned atoms were referred to as “opposite-direction”. High-resolution morphology images in Figure 6g,j show these two types of boundaries. The line spectra in Figure 6h,k reveal that at the same-direction boundary, the material exhibits metallic behavior in the H phase, whereas at the opposite-direction boundary, the material retains the insulating behavior of the T phase. Density functional theory (DFT) calculations of the band structure show that at the same-direction boundary, the UHB (upper Hubbard band) is absent in T-NbSe_2_, while at the opposite-direction boundary, the UHB shifts upward, and the bandgap between the UHB and LHB (lower Hubbard band) widens.

### 3.4. Atomic-Scale Etching and Nanostructure Customization

STM-based nano-etching has evolved through several key stages. In the 1980s, STM demonstrated nanoscale processing capabilities, enabling the etching of nanowires and conical structures on metal surfaces [85,86,87]. By the 1990s, electrochemical oxidation etching emerged as a breakthrough, with STM inducing local oxidation on hydrogen-passivated silicon, achieving precise nanostructure etching [88]. Simultaneously, thermal decomposition and electron beam etching advanced STM’s application under high-temperature and electron energy control [89]. As the technology progressed, STM expanded into electrochemical etching in liquid environments, enabling atomic-scale precision in more complex materials. By the late 1990s, STM achieved ultimate resolution with single-atom manipulation at ultralow temperatures, demonstrating atomic-level precision.

**Figure 7:**
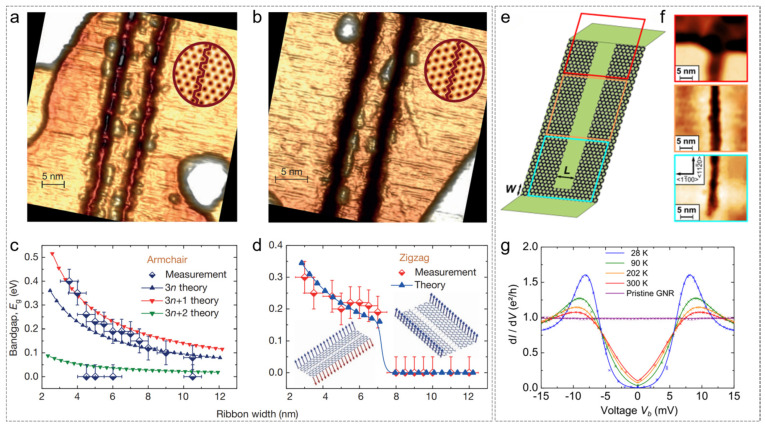
The customization of a graphene structure via an STM tip. (**a**–**d**) The customization of a graphene edge structure [65]. (**a**,**b**) STM images of GNRs with armchair and zigzag edges, respectively. (**c**,**d**) The bandgap measured as a function of ribbon width in armchair and zigzag ribbons. An STM study of a sidewall GNC. (**e**–**g**) Customized size for narrow constrictions [90]. (**e**) A schematic view of the graphene structure after the synthesis of a narrow constriction. (**f**) STM images of the top, center, and bottom part of the cut. (**g**) The differential conductance of the I–V curve at different temperatures. (**a**–**d**) Reprinted with permission from Ref. [65] from Springer Nature, copyright 2014. (**e**–**g**) Reprinted with permission from Ref. [90] from the American Physical Society, copyright 2016.

In 2014, researchers utilized STM-based etching techniques to create GNRs with precisely defined edge configurations and widths [65]. As shown in Figure 7a, the study investigated the effects of armchair and zigzag edge structures, as well as different widths, on the electrical conductivity and magnetism of graphene nanoribbons. Through a combination of theoretical and experimental work, it was found that when the width of the graphene nanoribbon was less than 7 nm, zigzag-edge ribbons exhibited a 200–300 meV bandgap, which arises from electron–electron interactions. However, when the width exceeded 8 nm, the bandgap disappeared. In contrast, the bandgap of armchair-edge nanoribbons (E_g_) was inversely proportional to their width (W).

In addition, using STM lithography, the researchers etched nanoscopic constrictions (GNCs) in graphene nanoribbons with atomic precision, achieving sizes on the order of a few nanometers [90]. The high bias and strong tunneling current from the STM locally broke carbon–carbon bonds, forming the constrictions while maintaining the integrity of the surrounding graphene. Following the introduction of the constriction, the current–voltage (I–V) curve became nonlinear, with a transport gap (Δ ≈ 10 meV) emerging at low-bias voltages. The height of the conductance peak exhibited an exponential decay with increasing temperature but remained clearly visible even at room temperature (300 K).

## 4. Summary and Perspectives

Combined with its exceptional precision for atomic-scale synthesis and manipulation, STM establishes a versatile platform for quantum research and the rational design of advanced materials—from molecular graphene to fractal nanostructures. Compared to alternative transfer methods, SPM-based techniques (particularly STM) enable nondestructive nanoscale manipulation with customizable geometry control while maintaining single-atom precision. Unlike AFM, which relies on tip–surface contact interactions, STM’s quantum tunneling-based noncontact operation not only permits atomic-resolution material restructuring but simultaneously provides real-time electronic characterization through current–voltage spectroscopy. This dual capability to both engineer matter at the fundamental quantum level and probe emergent physical phenomena enables: (1) the systematic construction of tailored nanoscale architectures with programmed electronic properties, and (2) the fundamental exploration of low-dimensional quantum systems through atom-by-atom fabrication. Certainly, the application of STM manipulation technology still faces significant challenges. Despite its strengths, several critical barriers persist. Currently, STM techniques are limited to small-sized samples and demand exceptionally high cleanliness standards for both specimens and the scanning environment. Furthermore, the process is vulnerable to interference from environmental vibrations, thermal drift, and electronic noise, undermining environmental stability during operations. While manual STM manipulation allows for precise atomic-level control, its low efficiency hampers the automation required for fabricating complex nanostructures and achieving large-scale assembly. Scaling these laboratory-proven techniques to practical industrial device fabrication introduces additional technical hurdles, particularly in maintaining precision and reproducibility at macroscopic scales. Addressing these limitations—automation capabilities, environmental robustness, and scalable manufacturing—remains pivotal for transitioning STM from a research tool to a mainstream nanofabrication platform.

This review has summarized the principal methods and recent advances in STM-based manipulation of two-dimensional materials, emphasizing its role in enabling precise structural control and property tuning. Looking ahead, the integration of STM with artificial intelligence (AI) holds great potential to transform nanofabrication. AI-driven approaches could facilitate autonomous manipulation, optimize experimental parameters in real time, and accelerate the design of functional nanodevices. As these interdisciplinary technologies evolve, STM is expected to play an increasingly pivotal role in the future of electronics, photonics, biomedicine, and nanoscale systems engineering.

## Data Availability

Not applicable.

## References

[1] Novoselov K.S., Geim A.K., Morozov S.V., Jiang D., Zhang Y., Dubonos S.V., Grigorieva I.V., Firsov A.A. (2004). Electric field effect in atomically thin carbon films. Science.

[2] Tan C., Cao X., Wu X.-J., He Q., Yang J., Zhang X., Chen J., Zhao W., Han S., Nam G.-H. (2017). Recent Advances in Ultrathin Two-Dimensional Nanomaterials. Chem. Rev..

[3] Zhang H. (2015). Ultrathin Two-Dimensional Nanomaterials. ACS Nano.

[4] Lin C.-Y., Wu J.-Y., Chiu Y.-H., Chang C.-P., Lin M.-F. (2014). Stacking-dependent magnetoelectronic properties in multilayer graphene. Phys. Rev. B.

[5] Shinjo K., Hirano M. (1993). Dynamics of friction: Superlubric state. Surf. Sci..

[6] Cao Y., Fatemi V., Fang S., Watanabe K., Taniguchi T., Kaxiras E., Jarillo-Herrero P. (2018). Unconventional superconductivity in magic-angle graphene superlattices. Nature.

[7] Zhao C., Tan C., Lien D.-H., Song X., Amani M., Hettick M., Nyein H.Y.Y., Yuan Z., Li L., Scott M.C. (2020). Evaporated tellurium thin films for p-type field-effect transistors and circuits. Nat. Nanotechnol..

[8] Koman V.B., Liu P., Kozawa D., Liu A.T., Cottrill A.L., Son Y., Lebron J.A., Strano M.S. (2018). Colloidal nanoelectronic state machines based on 2D materials for aerosolizable electronics. Nat. Nanotechnol..

[9] Zhu W., Low T., Wang H., Ye P., Duan X. (2019). Nanoscale electronic devices based on transition metal dichalcogenides. 2D Mater..

[10] Amani M., Tan C., Zhang G., Zhao C., Bullock J., Song X., Kim H., Shrestha V.R., Gao Y., Crozier K.B. (2018). Solution-Synthesized High-Mobility Tellurium Nanoflakes for Short-Wave Infrared Photodetectors. ACS Nano.

[11] Mak K.F., Shan J. (2016). Photonics and optoelectronics of 2D semiconductor transition metal dichalcogenides. Nat. Photonics.

[12] Huo C., Cai B., Yuan Z., Ma B., Zeng H. (2017). Two-Dimensional Metal Halide Perovskites: Theory, Synthesis, and Optoelectronics. Small Methods.

[13] Cheng Z., Cao R., Wei K., Yao Y., Liu X., Kang J., Dong J., Shi Z., Zhang H., Zhang X. (2021). 2D Materials Enabled Next-Generation Integrated Optoelectronics: From Fabrication to Applications. Adv. Sci..

[14] Liao M., Nicolini P., Du L., Yuan J., Wang S., Yu H., Tang J., Cheng P., Watanabe K., Taniguchi T. (2022). UItra-low friction and edge-pinning effect in large-lattice-mismatch van der Waals heterostructures. Nat. Mater..

[15] Sinclair R.C., Suter J.L., Coveney P.V. (2018). Graphene–graphene interactions: Friction, superlubricity, and exfoliation. Adv. Mater..

[16] Feng X., Kwon S., Park J.Y., Salmeron M. (2013). Superlubric Sliding of Graphene Nanoflakes on Graphene. ACS Nano.

[17] Zheng X., Gao L., Yao Q., Li Q., Zhang M., Xie X., Qiao S., Wang G., Ma T., Di Z. (2016). Robust ultra-low-friction state of graphene via moiré superlattice confinement. Nat. Commun..

[18] Li S., Li Q., Carpick R.W., Gumbsch P., Liu X.Z., Ding X., Sun J., Li J. (2016). The evolving quality of frictional contact with graphene. Nature.

[19] Choi J.S., Kim J.-S., Byun I.-S., Lee D.H., Lee M.J., Park B.H., Lee C., Yoon D., Cheong H., Lee K.H. (2011). Friction anisotropy–driven domain imaging on exfoliated monolayer graphene. Science.

[20] Thomson A., Chatterjee S., Sachdev S., Scheurer M.S. (2018). Triangular antiferromagnetism on the honeycomb lattice of twisted bilayer graphene. Phys. Rev. B.

[21] Geim A.K., Grigorieva I.V. (2013). Van der Waals heterostructures. Nature.

[22] Liao M., Wei Z., Du L., Wang Q., Tang J., Yu H., Wu F., Zhao J., Xu X., Han B. (2020). Precise control of the interlayer twist angle in large scale MoS_2_ homostructures. Nat. Commun..

[23] Wang B., Huang M., Kim N.Y., Cunning B.V., Huang Y., Qu D., Chen X., Jin S., Biswal M., Zhang X. (2017). Controlled Folding of Single Crystal Graphene. Nano Lett..

[24] Kim K., Yankowitz M., Fallahazad B., Kang S., Movva H.C.P., Huang S., Larentis S., Corbet C.M., Taniguchi T., Watanabe K. (2016). van der Waals Heterostructures with High Accuracy Rotational Alignment. Nano Lett..

[25] Yang Y., Li J., Yin J., Xu S., Mullan C., Taniguchi T., Watanabe K., Geim A.K., Novoselov K.S., Mishchenko A. (2020). In situ manipulation of van der Waals heterostructures for twistronics. Sci. Adv..

[26] Zhang Q., Hou Y., Zhang T., Xu Z., Huang Z., Yuan P., Jia L., Yang H., Huang Y., Ji W. (2021). Visualizing Spatial Evolution of Electron-Correlated Interface in Two-Dimensional Heterostructures. ACS Nano.

[27] Liao M., Wu Z.-W., Du L., Zhang T., Wei Z., Zhu J., Yu H., Tang J., Gu L., Xing Y. (2018). Twist angle-dependent conductivities across MoS_2_/graphene heterojunctions. Nat. Commun..

[28] Chang J.S., Kim S., Sung H.-J., Yeon J., Chang K.J., Li X., Kim S. (2018). Graphene Nanoribbons with Atomically Sharp Edges Produced by AFM Induced Self-Folding. Small.

[29] Wang D., Chen G., Li C., Cheng M., Yang W., Wu S., Xie G., Zhang J., Zhao J., Lu X. (2016). Thermally Induced Graphene Rotation on Hexagonal Boron Nitride. Phys. Rev. Lett..

[30] Chen P.Y., Zhang X.Q., Lai Y.Y., Lin E.C., Chen C.A., Guan S.Y., Chen J.J., Yang Z.H., Tseng Y.W., Gwo S. (2019). Tunable moiré superlattice of artificially twisted monolayers. Adv. Mater..

[31] Cao Y., Fatemi V., Demir A., Fang S., Tomarken S.L., Luo J.Y., Sanchez-Yamagishi J.D., Watanabe K., Taniguchi T., Kaxiras E. (2018). Correlated insulator behaviour at half-filling in magic-angle graphene superlattices. Nature.

[32] Bian K., Gerber C., Heinrich A.J., Müller D.J., Scheuring S., Jiang Y. (2021). Scanning probe microscopy. Nat. Rev. Methods Primers.

[33] Binnig G., Rohrer H., Gerber C., Weibel E.J.P.R.L. (1982). Surface studies by scanning tunneling microscopy. Phys. Rev. Lett..

[34] Huang Y., Sutter E., Shi N.N., Zheng J.B., Yang T.Z., Englund D., Gao H.J., Sutter P. (2015). Reliable Exfoliation of Large-Area High-Quality Flakes of Graphene and Other Two-Dimensional Materials. ACS Nano.

[35] Barron A.R., Khan M.R. (2008). Caorbon nanotubes: Opportunities and challenges. Adv. Mater. Process.

[36] Puente Santiago A.R., Fernandez-Delgado O., Gomez A., Ahsan M.A., Echegoyen L. (2021). Fullerenes as Key Components for Low-Dimensional (Photo)electrocatalytic Nanohybrid Materials. Angew. Chem. Int. Ed..

[37] Zheng X.T., Ananthanarayanan A., Luo K.Q., Chen P. (2015). Glowing Graphene Quantum Dots and Carbon Dots: Properties, Syntheses, and Biological Applications. Small.

[38] Ko W., Ma C., Nguyen G.D., Kolmer M., Li A.-P. (2019). Atomic-Scale Manipulation and In Situ Characterization with Scanning Tunneling Microscopy. Adv. Funct. Mater..

[39] Moller M., Jarvis S.P., Guerinet L., Sharp P., Woolley R., Rahe P., Moriarty P. (2017). Automated extraction of single H atoms with STM: Tip state dependency. Nanotechnology.

[40] Gomes K.K., Mar W., Ko W., Guinea F., Manoharan H.C. (2012). Designer Dirac fermions and topological phases in molecular graphene. Nature.

[41] Guo J., Meng X., Chen J., Peng J., Sheng J., Li X.-Z., Xu L., Shi J.-R., Wang E., Jiang Y. (2014). Real-space imaging of interfacial water with submolecular resolution. Nat. Mater..

[42] Lafferentz L., Ample F., Yu H., Hecht S., Joachim C., Grill L. (2009). Conductance of a Single Conjugated Polymer as a Continuous Function of Its Length. Science.

[43] Thupakula U., Bouju X., Castro-Esteban J., Dujardin E., Pena D., Joachim C. (2022). Planar bridging an atomically precise surface trench with a single molecular wire on an Au(111) surface. Chem. Phys. Lett..

[44] Eigler D.M., Schweizer E.K. (1990). Positioning single atoms with a scanning tunnelling microscope. Nature.

[45] Bartels L., Meyer G., Rieder K.H. (1997). Basic steps involved in the lateral manipulation of single CO molecules and rows of CO molecules. Chem. Phys. Lett..

[46] Wang Y., Wong D., Shytov A.V., Brar V.W., Choi S., Wu Q., Tsai H.-Z., Regan W., Zettl A., Kawakami R.K. (2013). Observing Atomic Collapse Resonances in Artificial Nuclei on Graphene. Science.

[47] Hla S.W., Meyer G., Rieder K.H.J.C. (2001). Inducing single-molecule chemical reactions with a UHV-STM: A new dimension for nano-science and technology. Phys. Rev. Lett..

[48] Nilius N., Wallis T.M., Ho W. (2002). Development of one-dimensional band structure in artificial gold chains. Science.

[49] Hirjibehedin C.F., Lutz C.P., Heinrich A.J. (2006). Spin coupling in engineered atomic structures. Science.

[50] Crommie M.F., Lutz C.P., Eigler D.M. (1993). Confinement of electrons to quantum corrals on a metal-surface. Science.

[51] Hla S.-W., Bartels L., Meyer G., Rieder K.-H. (2000). Inducing All Steps of a Chemical Reaction with the Scanning Tunneling Microscope Tip: Towards Single Molecule Engineering. Phys. Rev. Lett..

[52] Ma C., Liang L., Xiao Z., Puretzky A.A., Hong K., Lu W., Meunier V., Bernholc J., Li A.-P. (2017). Seamless Staircase Electrical Contact to Semiconducting Graphene Nanoribbons. Nano Lett..

[53] Friedrich N., Brandimarte P., Li J., Saito S., Yamaguchi S., Pozo I., Peña D., Frederiksen T., Garcia-Lekue A., Sánchez-Portal D. (2020). Magnetism of Topological Boundary States Induced by Boron Substitution in Graphene Nanoribbons. Phys. Rev. Lett..

[54] Zhang Q., Zhang Y., Hou Y., Xu R., Jia L., Huang Z., Hao X., Zhou J., Zhang T., Liu L. (2022). Nanoscale Control of One-Dimensional Confined States in Strongly Correlated Homojunctions. Nano Lett..

[55] Leicht P., Zielke L., Bouvron S., Moroni R., Voloshina E., Hammerschmidt L., Dedkov Y.S., Fonin M. (2014). In Situ Fabrication of Quasi-Free-Standing Epitaxial Graphene Nanoflakes On Gold. ACS Nano.

[56] Büch H., Rossi A., Forti S., Convertino D., Tozzini V., Coletti C. (2018). Superlubricity of epitaxial monolayer WS_2_ on graphene. Nano Res..

[57] Liu Y.-W., Hao C.-Y., He L. (2022). Tailoring the Energy Landscape of Graphene Nanostructures on Graphene and Manipulating Them Using Tilt Grain Boundaries. Phys. Rev. Appl..

[58] Chen H., Zhang X.-L., Zhang Y.-Y., Wang D., Bao D.-L., Que Y., Xiao W., Du S., Ouyang M., Pantelides S.T. (2019). Atomically precise, custom-design origami graphene nanostructures. Science.

[59] Li L.X., Liu R.P., Chen Z.W., Wang Q., Ma M.Z., Jing Q., Li G., Tian Y. (2006). Tearing, folding and deformation of a carbon-carbon sp^2^-bonded network. Carbon.

[60] Roy H.V., Kallinger C., Marsen B., Sattler K. (1998). Manipulation of graphitic sheets using a tunneling microscope. J. Appl. Phys..

[61] Jacobse P.H., Mangnus M.J.J., Zevenhuizen S.J.M., Swart I. (2018). Mapping the Conductance of Electronically Decoupled Graphene Nanoribbons. ACS Nano.

[62] Koch M., Ample F., Joachim C., Grill L. (2012). Voltage-dependent conductance of a single graphene nanoribbon. Nat. Nanotechnol..

[63] Wang S., Talirz L., Pignedoli C.A., Feng X., Müllen K., Fasel R., Ruffieux P. (2016). Giant edge state splitting at atomically precise graphene zigzag edges. Nat. Commun..

[64] Koós A.A., Vancsó P., Magda G.Z., Osváth Z., Kertész K., Dobrik G., Hwang C., Tapasztó L., Biró L.P. (2016). STM study of the MoS2 flakes grown on graphite: A model system for atomically clean 2D heterostructure interfaces. Carbon.

[65] Magda G.Z., Jin X., Hagymási I., Vancsó P., Osváth Z., Nemes-Incze P., Hwang C., Biró L.P., Tapasztó L. (2014). Room-temperature magnetic order on zigzag edges of narrow graphene nanoribbons. Nature.

[66] Dienwiebel M., Verhoeven G.S., Pradeep N., Frenken J.W.M., Heimberg J.A., Zandbergen H.W. (2004). Superlubricity of Graphite. Phys. Rev. Lett..

[67] Zheng Q., Jiang B., Liu S., Weng Y., Lu L., Xue Q., Zhu J., Jiang Q., Wang S., Peng L. (2008). Self-Retracting Motion of Graphite Microflakes. Phys. Rev. Lett..

[68] Ru G., Qi W., Tang K., Wei Y., Xue T. (2020). Interlayer friction and superlubricity in bilayer graphene and MoS_2_/MoSe_2_ van der Waals heterostructures. Tribol. Int..

[69] Gao F., Li D., Barreteau C., Brandbyge M. (2022). Proposal for All-Electrical Spin Manipulation and Detection for a Single Molecule on Boron-Substituted Graphene. Phys. Rev. Lett..

[70] Li J., Sanz S., Corso M., Choi D.J., Peña D., Frederiksen T., Pascual J.I. (2019). Single spin localization and manipulation in graphene open-shell nanostructures. Nat. Commun..

[71] Stühler R., Reis F., Müller T., Helbig T., Schwemmer T., Thomale R., Schäfer J., Claessen R. (2020). Tomonaga–Luttinger liquid in the edge channels of a quantum spin Hall insulator. Nat. Phys..

[72] Xia Y., Wang B., Zhang J., Jin Y., Tian H., Ho W., Xu H., Jin C., Xie M. (2020). Quantum confined Tomonaga–Luttinger liquid in Mo_6_Se_6_ nanowires converted from an epitaxial MoSe_2_ monolayer. Nano Lett..

[73] Zheng C., Zhang Q., Weber B., Ilatikhameneh H., Chen F., Sahasrabudhe H., Rahman R., Li S., Chen Z., Hellerstedt J. (2017). Direct observation of 2D electrostatics and ohmic contacts in template-grown graphene/WS_2_ heterostructures. ACS Nano.

[74] Chen Y., Zhang Y., Huang Z.-P., Jia L.-G., Liu L.-W., Yang H.-X., Zhang T., He L., Zhou J.-D., Huang Y. (2023). Visualization of Macroscopic Ising Superconducting State in Superconductor-Graphene Junctions. Adv. Quantum Technol..

[75] Hou B., Zhang Y., Zhang T., Wu J., Zhang Q., Han X., Huang Z., Chen Y., Ji H., Wang T. (2023). Multiple electronic phases coexisting under inhomogeneous strains in the correlated insulator. Adv. Sci..

[76] Guan W., Li K., Xiao Y., Li S.-Y., Pan A. (2022). Structural and electronic property engineering in graphene nanowrinkles via scanning tunneling microscopy. Phys. Rev. B.

[77] Chen H., Bao D.-L., Wang D., Que Y., Xiao W., Zhang Y.-Y., Sun J., Du S., Gao H.-J. (2020). Fabrication and manipulation of nanosized graphene homojunction with atomically-controlled boundaries. Nano Res..

[78] Wang D., Bao D.-L., Zheng Q., Wang C.-T., Wang S., Fan P., Mishra S., Tao L., Xiao Y., Huang L. (2023). Twisted bilayer zigzag-graphene nanoribbon junctions with tunable edge states. Nat. Commun..

[79] Zhang J., Liu J., Huang J.L., Kim P., Lieber C.M. (1996). Creation of nanocrystals through a solid-solid phase transition induced by an STM tip. Science.

[80] Bischoff F., Auwärter W., Barth J.V., Schiffrin A., Fuhrer M., Weber B. (2017). Nanoscale Phase Engineering of Niobium Diselenide. Chem. Mater..

[81] Song X., Liu L., Chen Y., Yang H., Huang Z., Hou B., Hou Y., Han X., Yang H., Zhang Q. (2022). Atomic-scale visualization of chiral charge density wave superlattices and their reversible switching. Nat. Commun..

[82] He Z., Poudel S.P., Stolz S., Wang T., Rossi A., Wang F., Mo S.-K., Weber-Bargioni A., Qiu Z.Q., Barraza-Lopez S. (2024). Synthesis and Polymorph Manipulation of FeSe_2_ Monolayers. Nano Lett..

[83] Ren Y.-N., Zhang M.-H., Zhou X.-F., Zheng Q., Ren H.-Y., He L. (2024). In situ creation and tailoring of interfacial quantum dots in graphene/transition metal dichalcogenide heterostructures. Phys. Rev. B.

[84] Chen Y., Dai Y.-X., Zhang Y., Zhang C., Zhou L., Jia L., Wang W., Han X., Yang H.X., Liu L. (2025). Nanoscale Polymorph Engineering of Metal-Correlated Insulator Junctions in Monolayer NbSe_2_. ACS Nano.

[85] Ringger M., Hidber H.R., Schlögl R., Oelhafen P., Güntherodt H.J. (1985). Nanometer lithography with the scanning tunneling microscope. Appl. Phys. Lett..

[86] Wiesendanger R., Eng L., Hidber H.R., Oelhafen P., Rosenthaler L., Staufer U., Güntherodt H.J. (1987). Local tunneling barrier height images obtained with the scanning tunneling microscope. Surf. Sci..

[87] Tománek D., Louie S.G., Mamin H.J., Abraham D.W., Thomson R.E., Ganz E., Clarke J. (1987). Theory and observation of highly asymmetric atomic structure in scanning-tunneling-microscopy images of graphite. Phys. Rev. B.

[88] Dagata J.A., Schneir J., Harary H.H., Evans C.J., Postek M.T., Bennett J. (1990). Modification of hydrogen-passivated silicon by a scanning tunneling microscope operating in air. Appl. Phys. Lett..

[89] Li N.L.N., Yoshinobu T.Y.T., Iwasaki H.I.H. (1998). Low energy electron beam stimulated surface reaction: Selective etching of SiO_2_/Si using scanning tunneling microscope. Jpn. J. Appl. Phys..

[90] Baringhaus J., Settnes M., Aprojanz J., Power S.R., Jauho A.P., Tegenkamp C. (2016). Electron Interference in Ballistic Graphene Nanoconstrictions. Phys. Rev. Lett..

